# Supplementary material for: Community diversity is associated with intra-species genetic diversity and gene loss in the human gut microbiome
Source: eLife. 2023 Feb 9;12:e78530. doi: 10.7554/eLife.78530 (PMC9977275; doi:10.7554/eLife.78530)
Supplement: Supplementary file 2. [file elife-78530-supp2.docx]

GLMMs and GAMs summary

Naima Madi

19/10/2022

## 1. GAMs with polymorphism rate at synonymous sites (S) in a focal species as a function of community diversity

### 1.1. Polymorphism rate (S) as a function of shannon diversity

##
## Family: Beta regression(83.127)
## Link function: logit
##
## Formula:
## polymorphism_rate ~ s(species_name, Shannon_alpha_diversity,
## bs = "fs") + s(sample_id, bs = "re") + s(subject_id, bs = "re") +
## s(Shannon_alpha_diversity) + s(total_reads_orig)
##
## Parametric coefficients:
## Estimate Std. Error z value Pr(>|z|)
## (Intercept) -5.50462 0.06222 -88.47 <2e-16 ***
## ---
## Signif. codes: 0 '***' 0.001 '**' 0.01 '*' 0.05 '.' 0.1 ' ' 1
##
## Approximate significance of smooth terms:
## edf Ref.df Chi.sq p-value
## s(species_name,Shannon_alpha_diversity) 94.2839 657.000 3045.219 < 2e-16 ***
## s(sample_id) 0.1071 464.000 0.064 1.000000
## s(subject_id) 126.8345 248.000 341.220 < 2e-16 ***
## s(Shannon_alpha_diversity) 1.0654 1.110 4.687 0.031412 *
## s(total_reads_orig) 2.1916 2.622 15.605 0.000883 ***
## ---
## Signif. codes: 0 '***' 0.001 '**' 0.01 '*' 0.05 '.' 0.1 ' ' 1
##
## R-sq.(adj) = 0.198 Deviance explained = 33.2%
## -REML = -44506 Scale est. = 1 n = 8324

### 1.2. Polymorphism rate (S) as a function of species richness (estimated on all data)

##
## Family: Beta regression(82.337)
## Link function: logit
##
## Formula:
## polymorphism_rate ~ s(species_richness) + s(total_reads_orig) +
## s(species_name, species_richness, bs = "fs") + s(sample_id,
## bs = "re") + s(subject_id, bs = "re")
##
## Parametric coefficients:
## Estimate Std. Error z value Pr(>|z|)
## (Intercept) -5.50343 0.06384 -86.21 <2e-16 ***
## ---
## Signif. codes: 0 '***' 0.001 '**' 0.01 '*' 0.05 '.' 0.1 ' ' 1
##
## Approximate significance of smooth terms:
## edf Ref.df Chi.sq p-value
## s(species_richness) 2.3921 2.897 10.596 0.0172 *
## s(total_reads_orig) 2.6499 3.180 29.839 2.32e-06 ***
## s(species_name,species_richness) 74.3303 653.000 2899.471 < 2e-16 ***
## s(sample_id) 0.1199 464.000 0.069 1.0000
## s(subject_id) 129.6311 248.000 355.976 < 2e-16 ***
## ---
## Signif. codes: 0 '***' 0.001 '**' 0.01 '*' 0.05 '.' 0.1 ' ' 1
##
## R-sq.(adj) = 0.191 Deviance explained = 32.3%
## -REML = -44490 Scale est. = 1 n = 8324

### 1.3. Polymorphism rate (S) as a function of species richness (estimated on rarefied data)

##
## Family: Beta regression(81.16)
## Link function: logit
##
## Formula:
## polymorphism_rate ~ s(species_richness) + s(total_reads_orig) +
## s(species_name, species_richness, bs = "fs") + s(sample_id,
## bs = "re") + s(subject_id, bs = "re")
##
## Parametric coefficients:
## Estimate Std. Error z value Pr(>|z|)
## (Intercept) -5.49604 0.06379 -86.16 <2e-16 ***
## ---
## Signif. codes: 0 '***' 0.001 '**' 0.01 '*' 0.05 '.' 0.1 ' ' 1
##
## Approximate significance of smooth terms:
## edf Ref.df Chi.sq p-value
## s(species_richness) 2.68466 3.292 20.199 0.000263 ***
## s(total_reads_orig) 1.97641 2.344 8.043 0.020662 *
## s(species_name,species_richness) 64.97097 664.000 2823.442 < 2e-16 ***
## s(sample_id) 0.03536 434.000 0.021 1.000000
## s(subject_id) 121.68267 234.000 319.502 < 2e-16 ***
## ---
## Signif. codes: 0 '***' 0.001 '**' 0.01 '*' 0.05 '.' 0.1 ' ' 1
##
## R-sq.(adj) = 0.19 Deviance explained = 32.2%
## -REML = -43612 Scale est. = 1 n = 8153

## 2. GAMs with polymorphism rate at synonymous sites in a focal species as a function of shannon diversity at higher taxonomic levels

### 2.1. Polymorphism (S) rate as a function of phylum shannon diversity

##
## Family: Beta regression(82.102)
## Link function: logit
##
## Formula:
## polymorphism_rate ~ s(species_name, phyla_shannon, bs = "fs") +
## s(sample_id, bs = "re") + s(subject_id, bs = "re") + s(phyla_shannon) +
## s(total_reads_orig)
##
## Parametric coefficients:
## Estimate Std. Error z value Pr(>|z|)
## (Intercept) -5.50443 0.06413 -85.83 <2e-16 ***
## ---
## Signif. codes: 0 '***' 0.001 '**' 0.01 '*' 0.05 '.' 0.1 ' ' 1
##
## Approximate significance of smooth terms:
## edf Ref.df Chi.sq p-value
## s(species_name,phyla_shannon) 63.0641 667.000 2845.917 < 2e-16 ***
## s(sample_id) 0.0525 464.000 0.030 1.000000
## s(subject_id) 135.0904 248.000 391.928 < 2e-16 ***
## s(phyla_shannon) 1.0008 1.001 0.399 0.528475
## s(total_reads_orig) 2.2930 2.750 20.019 0.000224 ***
## ---
## Signif. codes: 0 '***' 0.001 '**' 0.01 '*' 0.05 '.' 0.1 ' ' 1
##
## R-sq.(adj) = 0.189 Deviance explained = 32%
## -REML = -44485 Scale est. = 1 n = 8324

### 2.2. Polymorphism rate (S) as a function of class shannon diversity

##
## Family: Beta regression(82.102)
## Link function: logit
##
## Formula:
## polymorphism_rate ~ s(species_name, class_shannon, bs = "fs") +
## s(sample_id, bs = "re") + s(subject_id, bs = "re") + s(class_shannon) +
## s(total_reads_orig)
##
## Parametric coefficients:
## Estimate Std. Error z value Pr(>|z|)
## (Intercept) -5.50443 0.06413 -85.83 <2e-16 ***
## ---
## Signif. codes: 0 '***' 0.001 '**' 0.01 '*' 0.05 '.' 0.1 ' ' 1
##
## Approximate significance of smooth terms:
## edf Ref.df Chi.sq p-value
## s(species_name,class_shannon) 63.07154 668.000 2846.265 < 2e-16 ***
## s(sample_id) 0.05227 464.000 0.030 1.000000
## s(subject_id) 135.07734 248.000 391.858 < 2e-16 ***
## s(class_shannon) 1.00078 1.001 0.418 0.518425
## s(total_reads_orig) 2.29239 2.749 19.996 0.000227 ***
## ---
## Signif. codes: 0 '***' 0.001 '**' 0.01 '*' 0.05 '.' 0.1 ' ' 1
##
## R-sq.(adj) = 0.189 Deviance explained = 32%
## -REML = -44485 Scale est. = 1 n = 8324

### 2.3. Polymorphism rate (S) as a function of order shannon diversity

##
## Family: Beta regression(82.095)
## Link function: logit
##
## Formula:
## polymorphism_rate ~ s(species_name, order_shannon, bs = "fs") +
## s(sample_id, bs = "re") + s(subject_id, bs = "re") + s(order_shannon) +
## s(total_reads_orig)
##
## Parametric coefficients:
## Estimate Std. Error z value Pr(>|z|)
## (Intercept) -5.50422 0.06409 -85.88 <2e-16 ***
## ---
## Signif. codes: 0 '***' 0.001 '**' 0.01 '*' 0.05 '.' 0.1 ' ' 1
##
## Approximate significance of smooth terms:
## edf Ref.df Chi.sq p-value
## s(species_name,order_shannon) 63.05687 667.000 2836.488 < 2e-16 ***
## s(sample_id) 0.03421 464.000 0.019 1.000000
## s(subject_id) 134.82575 248.000 389.563 < 2e-16 ***
## s(order_shannon) 1.00290 1.005 0.732 0.394604
## s(total_reads_orig) 2.28820 2.744 19.891 0.000235 ***
## ---
## Signif. codes: 0 '***' 0.001 '**' 0.01 '*' 0.05 '.' 0.1 ' ' 1
##
## R-sq.(adj) = 0.189 Deviance explained = 32%
## -REML = -44485 Scale est. = 1 n = 8324

### 2.4. Polymorphism rate (S) as a function of family shannon diversity

##
## Family: Beta regression(82.6)
## Link function: logit
##
## Formula:
## polymorphism_rate ~ s(species_name, family_shannon, bs = "fs") +
## s(sample_id, bs = "re") + s(subject_id, bs = "re") + s(family_shannon) +
## s(total_reads_orig)
##
## Parametric coefficients:
## Estimate Std. Error z value Pr(>|z|)
## (Intercept) -5.50114 0.06373 -86.32 <2e-16 ***
## ---
## Signif. codes: 0 '***' 0.001 '**' 0.01 '*' 0.05 '.' 0.1 ' ' 1
##
## Approximate significance of smooth terms:
## edf Ref.df Chi.sq p-value
## s(species_name,family_shannon) 81.6475 665.000 2929.823 < 2e-16 ***
## s(sample_id) 0.0644 464.000 0.038 1.000000
## s(subject_id) 128.9861 248.000 351.108 < 2e-16 ***
## s(family_shannon) 1.0027 1.005 4.676 0.030850 *
## s(total_reads_orig) 2.1864 2.617 15.737 0.000807 ***
## ---
## Signif. codes: 0 '***' 0.001 '**' 0.01 '*' 0.05 '.' 0.1 ' ' 1
##
## R-sq.(adj) = 0.192 Deviance explained = 32.6%
## -REML = -44497 Scale est. = 1 n = 8324

### 2.5. Polymorphism rate (S) as a function of genus shannon diversity

##
## Family: Beta regression(82.709)
## Link function: logit
##
## Formula:
## polymorphism_rate ~ s(species_name, genus_shannon, bs = "fs") +
## s(sample_id, bs = "re") + s(subject_id, bs = "re") + s(genus_shannon) +
## s(total_reads_orig)
##
## Parametric coefficients:
## Estimate Std. Error z value Pr(>|z|)
## (Intercept) -5.5000 0.0636 -86.48 <2e-16 ***
## ---
## Signif. codes: 0 '***' 0.001 '**' 0.01 '*' 0.05 '.' 0.1 ' ' 1
##
## Approximate significance of smooth terms:
## edf Ref.df Chi.sq p-value
## s(species_name,genus_shannon) 84.64871 665.000 2937.879 < 2e-16 ***
## s(sample_id) 0.04288 464.000 0.026 1.00000
## s(subject_id) 125.68401 248.000 330.285 < 2e-16 ***
## s(genus_shannon) 1.00319 1.006 6.506 0.01093 *
## s(total_reads_orig) 2.17477 2.603 15.552 0.00086 ***
## ---
## Signif. codes: 0 '***' 0.001 '**' 0.01 '*' 0.05 '.' 0.1 ' ' 1
##
## R-sq.(adj) = 0.193 Deviance explained = 32.7%
## -REML = -44502 Scale est. = 1 n = 8324

## 3. GAMs with polymorphism rate (at synonymous sites) in a focal species as a function of richness at higher taxonomic levels

### 3.1. Polymorphism rate (S) as a function of phylum richness

##
## Family: Beta regression(82.85)
## Link function: logit
##
## Formula:
## polymorphism_rate ~ s(species_name, phyla_nb, bs = "fs", k = 5) +
## s(sample_id, bs = "re", k = 5) + s(subject_id, bs = "re",
## k = 5) + s(phyla_nb, k = 5) + s(total_reads_orig, k = 5)
##
## Parametric coefficients:
## Estimate Std. Error z value Pr(>|z|)
## (Intercept) -5.50599 0.06421 -85.75 <2e-16 ***
## ---
## Signif. codes: 0 '***' 0.001 '**' 0.01 '*' 0.05 '.' 0.1 ' ' 1
##
## Approximate significance of smooth terms:
## edf Ref.df Chi.sq p-value
## s(species_name,phyla_nb) 84.18112 332.000 3039.620 < 2e-16 ***
## s(sample_id) 0.05118 464.000 0.029 1.00000
## s(subject_id) 129.94503 248.000 356.246 < 2e-16 ***
## s(phyla_nb) 1.00193 1.003 7.517 0.00614 **
## s(total_reads_orig) 2.19187 2.550 24.352 2.07e-05 ***
## ---
## Signif. codes: 0 '***' 0.001 '**' 0.01 '*' 0.05 '.' 0.1 ' ' 1
##
## R-sq.(adj) = 0.196 Deviance explained = 32.8%
## -REML = -44497 Scale est. = 1 n = 8324

### 3.2. Polymorphism rate (S) as a function of class richness

##
## Family: Beta regression(82.57)
## Link function: logit
##
## Formula:
## polymorphism_rate ~ s(species_name, class_nb, bs = "fs", k = 5) +
## s(sample_id, bs = "re", k = 5) + s(subject_id, bs = "re",
## k = 5) + s(class_nb, k = 5) + s(total_reads_orig, k = 5)
##
## Parametric coefficients:
## Estimate Std. Error z value Pr(>|z|)
## (Intercept) -5.50526 0.06414 -85.83 <2e-16 ***
## ---
## Signif. codes: 0 '***' 0.001 '**' 0.01 '*' 0.05 '.' 0.1 ' ' 1
##
## Approximate significance of smooth terms:
## edf Ref.df Chi.sq p-value
## s(species_name,class_nb) 7.888e+01 336.000 2985.657 < 2e-16 ***
## s(sample_id) 3.942e-03 464.000 0.002 1.0000
## s(subject_id) 1.317e+02 248.000 368.973 < 2e-16 ***
## s(class_nb) 1.003e+00 1.005 6.501 0.0109 *
## s(total_reads_orig) 2.254e+00 2.618 26.160 1.23e-05 ***
## ---
## Signif. codes: 0 '***' 0.001 '**' 0.01 '*' 0.05 '.' 0.1 ' ' 1
##
## R-sq.(adj) = 0.194 Deviance explained = 32.5%
## -REML = -44492 Scale est. = 1 n = 8324

### 3.3. Polymorphism rate (S) as a function of order richness

##
## Family: Beta regression(82.285)
## Link function: logit
##
## Formula:
## polymorphism_rate ~ s(species_name, order_nb, bs = "fs", k = 5) +
## s(sample_id, bs = "re", k = 5) + s(subject_id, bs = "re",
## k = 5) + s(order_nb, k = 5) + s(total_reads_orig, k = 5)
##
## Parametric coefficients:
## Estimate Std. Error z value Pr(>|z|)
## (Intercept) -5.50518 0.06406 -85.94 <2e-16 ***
## ---
## Signif. codes: 0 '***' 0.001 '**' 0.01 '*' 0.05 '.' 0.1 ' ' 1
##
## Approximate significance of smooth terms:
## edf Ref.df Chi.sq p-value
## s(species_name,order_nb) 72.524 337.000 2913.982 < 2e-16 ***
## s(sample_id) 0.039 464.000 0.022 1.0000
## s(subject_id) 132.918 248.000 372.550 < 2e-16 ***
## s(order_nb) 1.009 1.015 5.628 0.0181 *
## s(total_reads_orig) 2.214 2.576 25.006 1.01e-05 ***
## ---
## Signif. codes: 0 '***' 0.001 '**' 0.01 '*' 0.05 '.' 0.1 ' ' 1
##
## R-sq.(adj) = 0.19 Deviance explained = 32.2%
## -REML = -44488 Scale est. = 1 n = 8324

### 3.4. Polymorphism rate (S) as a function of family richness

##
## Family: Beta regression(82.251)
## Link function: logit
##
## Formula:
## polymorphism_rate ~ s(species_name, family_nb, bs = "fs", k = 5) +
## s(sample_id, bs = "re", k = 5) + s(subject_id, bs = "re",
## k = 5) + s(family_nb, k = 5) + s(total_reads_orig, k = 5)
##
## Parametric coefficients:
## Estimate Std. Error z value Pr(>|z|)
## (Intercept) -5.50380 0.06389 -86.15 <2e-16 ***
## ---
## Signif. codes: 0 '***' 0.001 '**' 0.01 '*' 0.05 '.' 0.1 ' ' 1
##
## Approximate significance of smooth terms:
## edf Ref.df Chi.sq p-value
## s(species_name,family_nb) 71.93665 338.000 2902.469 < 2e-16 ***
## s(sample_id) 0.03462 464.000 0.020 1.00000
## s(subject_id) 130.42838 248.000 354.350 < 2e-16 ***
## s(family_nb) 1.00032 1.001 9.753 0.00179 **
## s(total_reads_orig) 2.24867 2.615 27.497 3.37e-06 ***
## ---
## Signif. codes: 0 '***' 0.001 '**' 0.01 '*' 0.05 '.' 0.1 ' ' 1
##
## R-sq.(adj) = 0.191 Deviance explained = 32.2%
## -REML = -44490 Scale est. = 1 n = 8324

### 3.5. Polymorphism rate (S) as a function of genus richness

##
## Family: Beta regression(82.453)
## Link function: logit
##
## Formula:
## polymorphism_rate ~ s(species_name, genus_nb, bs = "fs", k = 5) +
## s(sample_id, bs = "re", k = 5) + s(subject_id, bs = "re",
## k = 5) + s(genus_nb, k = 5) + s(total_reads_orig, k = 5)
##
## Parametric coefficients:
## Estimate Std. Error z value Pr(>|z|)
## (Intercept) -5.50417 0.06381 -86.26 <2e-16 ***
## ---
## Signif. codes: 0 '***' 0.001 '**' 0.01 '*' 0.05 '.' 0.1 ' ' 1
##
## Approximate significance of smooth terms:
## edf Ref.df Chi.sq p-value
## s(species_name,genus_nb) 78.66673 338.000 2923.286 < 2e-16 ***
## s(sample_id) 0.01746 464.000 0.010 1.00000
## s(subject_id) 129.82405 248.000 353.898 < 2e-16 ***
## s(genus_nb) 1.00718 1.012 8.095 0.00448 **
## s(total_reads_orig) 2.28861 2.659 28.260 2.42e-06 ***
## ---
## Signif. codes: 0 '***' 0.001 '**' 0.01 '*' 0.05 '.' 0.1 ' ' 1
##
## R-sq.(adj) = 0.193 Deviance explained = 32.4%
## -REML = -44492 Scale est. = 1 n = 8324

## 4. GAMs with polymorphism rate at non synonymous sites (NS) in a focal species as a function of community diversity

### 4.1. Polymorphism rate (NS) as a function of shannon diversity

##
## Family: Beta regression(854.674)
## Link function: logit
##
## Formula:
## polymorphism_nonsyn ~ s(species, alpha_div, bs = "fs") + s(sample_id,
## bs = "re") + s(subject, bs = "re") + s(alpha_div) + s(total_reads_orig)
##
## Parametric coefficients:
## Estimate Std. Error z value Pr(>|z|)
## (Intercept) -7.40965 0.05846 -126.8 <2e-16 ***
## ---
## Signif. codes: 0 '***' 0.001 '**' 0.01 '*' 0.05 '.' 0.1 ' ' 1
##
## Approximate significance of smooth terms:
## edf Ref.df Chi.sq p-value
## s(species,alpha_div) 121.6380 668.000 3426.332 <2e-16 ***
## s(sample_id) 0.3785 464.000 0.313 0.989
## s(subject) 145.1280 248.000 468.255 <2e-16 ***
## s(alpha_div) 1.0336 1.056 0.070 0.842
## s(total_reads_orig) 3.2545 3.923 92.304 <2e-16 ***
## ---
## Signif. codes: 0 '***' 0.001 '**' 0.01 '*' 0.05 '.' 0.1 ' ' 1
##
## R-sq.(adj) = 0.248 Deviance explained = 33.6%
## -REML = -56566 Scale est. = 1 n = 8324

### 4.2. Polymorphism rate (NS) as a function of species richness (all data)

##
## Family: Beta regression(848.533)
## Link function: logit
##
## Formula:
## polymorphism_nonsyn ~ s(richness) + s(total_reads_orig) + s(species,
## richness, bs = "fs") + s(sample_id, bs = "re") + s(subject,
## bs = "re")
##
## Parametric coefficients:
## Estimate Std. Error z value Pr(>|z|)
## (Intercept) -7.40651 0.05218 -141.9 <2e-16 ***
## ---
## Signif. codes: 0 '***' 0.001 '**' 0.01 '*' 0.05 '.' 0.1 ' ' 1
##
## Approximate significance of smooth terms:
## edf Ref.df Chi.sq p-value
## s(richness) 3.5898 4.301 8.238 0.0928 .
## s(total_reads_orig) 3.6766 4.421 109.187 <2e-16 ***
## s(species,richness) 123.6962 667.000 3233.935 <2e-16 ***
## s(sample_id) 0.1284 464.000 0.103 0.9963
## s(subject) 140.2825 248.000 432.162 <2e-16 ***
## ---
## Signif. codes: 0 '***' 0.001 '**' 0.01 '*' 0.05 '.' 0.1 ' ' 1
##
## R-sq.(adj) = 0.243 Deviance explained = 33.1%
## -REML = -56534 Scale est. = 1 n = 8324

### 4.3. Polymorphism rate (NS) as a function of species richness (rarefied data)

##
## Family: Beta regression(851.252)
## Link function: logit
##
## Formula:
## polymorphism_nonsyn ~ s(richness_rare) + s(total_reads_orig) +
## s(species, richness_rare, bs = "fs") + s(sample_id, bs = "re") +
## s(subject, bs = "re")
##
## Parametric coefficients:
## Estimate Std. Error z value Pr(>|z|)
## (Intercept) -7.40824 0.06345 -116.8 <2e-16 ***
## ---
## Signif. codes: 0 '***' 0.001 '**' 0.01 '*' 0.05 '.' 0.1 ' ' 1
##
## Approximate significance of smooth terms:
## edf Ref.df Chi.sq p-value
## s(richness_rare) 1.5383 1.807 1.985 0.257
## s(total_reads_orig) 3.4215 4.124 92.685 <2e-16 ***
## s(species,richness_rare) 117.7944 665.000 3371.510 <2e-16 ***
## s(sample_id) 0.2617 464.000 0.220 0.981
## s(subject) 143.7846 248.000 445.570 <2e-16 ***
## ---
## Signif. codes: 0 '***' 0.001 '**' 0.01 '*' 0.05 '.' 0.1 ' ' 1
##
## R-sq.(adj) = 0.244 Deviance explained = 33.3%
## -REML = -56567 Scale est. = 1 n = 8324

## 5. GAMs with polymorphism rate (at non synonymous sites) in a focal species as a function of shannon diversity at higher taxonomic levels

### 5.1. Polymorphism rate (NS) as a function of phylum shannon diversity

##
## Family: Beta regression(850.356)
## Link function: logit
##
## Formula:
## polymorphism_nonsyn ~ s(species, phyla_shannon, bs = "fs") +
## s(sample_id, bs = "re") + s(subject, bs = "re") + s(phyla_shannon) +
## s(total_reads_orig)
##
## Parametric coefficients:
## Estimate Std. Error z value Pr(>|z|)
## (Intercept) -7.41389 0.06201 -119.6 <2e-16 ***
## ---
## Signif. codes: 0 '***' 0.001 '**' 0.01 '*' 0.05 '.' 0.1 ' ' 1
##
## Approximate significance of smooth terms:
## edf Ref.df Chi.sq p-value
## s(species,phyla_shannon) 103.872 668.000 3269.572 <2e-16 ***
## s(sample_id) 0.135 464.000 0.109 0.995
## s(subject) 150.384 248.000 511.372 <2e-16 ***
## s(phyla_shannon) 1.033 1.058 1.141 0.284
## s(total_reads_orig) 3.310 3.997 97.817 <2e-16 ***
## ---
## Signif. codes: 0 '***' 0.001 '**' 0.01 '*' 0.05 '.' 0.1 ' ' 1
##
## R-sq.(adj) = 0.241 Deviance explained = 33.1%
## -REML = -56564 Scale est. = 1 n = 8324

### 5.2. Polymorphism rate (NS) as a function of class shannon diversity

##
## Family: Beta regression(851.796)
## Link function: logit
##
## Formula:
## polymorphism_nonsyn ~ s(species, class_shannon, bs = "fs") +
## s(sample_id, bs = "re") + s(subject, bs = "re") + s(class_shannon) +
## s(total_reads_orig)
##
## Parametric coefficients:
## Estimate Std. Error z value Pr(>|z|)
## (Intercept) -7.40564 0.06189 -119.7 <2e-16 ***
## ---
## Signif. codes: 0 '***' 0.001 '**' 0.01 '*' 0.05 '.' 0.1 ' ' 1
##
## Approximate significance of smooth terms:
## edf Ref.df Chi.sq p-value
## s(species,class_shannon) 109.85392 668.000 3280.865 <2e-16 ***
## s(sample_id) 0.08542 464.000 0.069 0.995
## s(subject) 151.01909 248.000 514.122 <2e-16 ***
## s(class_shannon) 2.00624 2.457 2.804 0.286
## s(total_reads_orig) 3.28034 3.960 103.538 <2e-16 ***
## ---
## Signif. codes: 0 '***' 0.001 '**' 0.01 '*' 0.05 '.' 0.1 ' ' 1
##
## R-sq.(adj) = 0.24 Deviance explained = 33.4%
## -REML = -56558 Scale est. = 1 n = 8324

### 5.3. Polymorphism rate (NS) as a function of order shannon diversity

##
## Family: Beta regression(852.236)
## Link function: logit
##
## Formula:
## polymorphism_nonsyn ~ s(species, order_shannon, bs = "fs") +
## s(sample_id, bs = "re") + s(subject, bs = "re") + s(order_shannon) +
## s(total_reads_orig)
##
## Parametric coefficients:
## Estimate Std. Error z value Pr(>|z|)
## (Intercept) -7.41338 0.06557 -113.1 <2e-16 ***
## ---
## Signif. codes: 0 '***' 0.001 '**' 0.01 '*' 0.05 '.' 0.1 ' ' 1
##
## Approximate significance of smooth terms:
## edf Ref.df Chi.sq p-value
## s(species,order_shannon) 91.9823 668.000 3291.619 <2e-16 ***
## s(sample_id) 0.1375 464.000 0.109 0.997
## s(subject) 151.2407 248.000 513.477 <2e-16 ***
## s(order_shannon) 2.4651 2.999 5.446 0.138
## s(total_reads_orig) 3.2664 3.942 102.025 <2e-16 ***
## ---
## Signif. codes: 0 '***' 0.001 '**' 0.01 '*' 0.05 '.' 0.1 ' ' 1
##
## R-sq.(adj) = 0.242 Deviance explained = 33.2%
## -REML = -56582 Scale est. = 1 n = 8324

### 5.4. Polymorphism rate (NS) as a function of family shannon diversity

##
## Family: Beta regression(851.323)
## Link function: logit
##
## Formula:
## polymorphism_nonsyn ~ s(species, family_shannon, bs = "fs") +
## s(sample_id, bs = "re") + s(subject, bs = "re") + s(family_shannon) +
## s(total_reads_orig)
##
## Parametric coefficients:
## Estimate Std. Error z value Pr(>|z|)
## (Intercept) -7.41676 0.06363 -116.6 <2e-16 ***
## ---
## Signif. codes: 0 '***' 0.001 '**' 0.01 '*' 0.05 '.' 0.1 ' ' 1
##
## Approximate significance of smooth terms:
## edf Ref.df Chi.sq p-value
## s(species,family_shannon) 90.1645 665.000 3333.078 <2e-16 ***
## s(sample_id) 0.1478 464.000 0.122 0.988
## s(subject) 146.9384 248.000 477.546 <2e-16 ***
## s(family_shannon) 1.0031 1.006 0.041 0.842
## s(total_reads_orig) 3.2506 3.922 90.611 <2e-16 ***
## ---
## Signif. codes: 0 '***' 0.001 '**' 0.01 '*' 0.05 '.' 0.1 ' ' 1
##
## R-sq.(adj) = 0.244 Deviance explained = 33%
## -REML = -56588 Scale est. = 1 n = 8324

### 5.5. Polymorphism rate (NS) as a function of genus shannon diversity

##
## Family: Beta regression(855.56)
## Link function: logit
##
## Formula:
## polymorphism_nonsyn ~ s(species, genus_shannon, bs = "fs") +
## s(sample_id, bs = "re") + s(subject, bs = "re") + s(genus_shannon) +
## s(total_reads_orig)
##
## Parametric coefficients:
## Estimate Std. Error z value Pr(>|z|)
## (Intercept) -7.41443 0.06309 -117.5 <2e-16 ***
## ---
## Signif. codes: 0 '***' 0.001 '**' 0.01 '*' 0.05 '.' 0.1 ' ' 1
##
## Approximate significance of smooth terms:
## edf Ref.df Chi.sq p-value
## s(species,genus_shannon) 105.3751 668.000 3402.444 <2e-16 ***
## s(sample_id) 0.1687 464.000 0.143 0.977
## s(subject) 145.6475 248.000 466.025 <2e-16 ***
## s(genus_shannon) 1.0056 1.009 0.028 0.871
## s(total_reads_orig) 3.2527 3.924 91.715 <2e-16 ***
## ---
## Signif. codes: 0 '***' 0.001 '**' 0.01 '*' 0.05 '.' 0.1 ' ' 1
##
## R-sq.(adj) = 0.247 Deviance explained = 33.5%
## -REML = -56592 Scale est. = 1 n = 8324

## 6. GAMs with polymorphism rate (at non synonymous sites) in a focal species as a function of richness at higher taxonomic levels

### 6.1. Polymorphism rate (NS) as a function of phylum richness

##
## Family: Beta regression(849.927)
## Link function: logit
##
## Formula:
## polymorphism_nonsyn ~ s(species, phyla_nb, k = 5, bs = "fs") +
## s(sample_id, k = 5, bs = "re") + s(subject, k = 5, bs = "re") +
## s(phyla_nb, k = 5) + s(total_reads_orig, k = 5)
##
## Parametric coefficients:
## Estimate Std. Error z value Pr(>|z|)
## (Intercept) -7.41984 0.06468 -114.7 <2e-16 ***
## ---
## Signif. codes: 0 '***' 0.001 '**' 0.01 '*' 0.05 '.' 0.1 ' ' 1
##
## Approximate significance of smooth terms:
## edf Ref.df Chi.sq p-value
## s(species,phyla_nb) 77.4818 332.000 3399.932 <2e-16 ***
## s(sample_id) 0.1337 464.000 0.107 0.9969
## s(subject) 144.3327 248.000 456.184 <2e-16 ***
## s(phyla_nb) 1.0038 1.006 13.105 0.0003 ***
## s(total_reads_orig) 2.9432 3.328 107.353 <2e-16 ***
## ---
## Signif. codes: 0 '***' 0.001 '**' 0.01 '*' 0.05 '.' 0.1 ' ' 1
##
## R-sq.(adj) = 0.246 Deviance explained = 32.7%
## -REML = -56594 Scale est. = 1 n = 8324

### 6.2. Polymorphism rate (NS) as a function of class richness

##
## Family: Beta regression(848.574)
## Link function: logit
##
## Formula:
## polymorphism_nonsyn ~ s(species, class_nb, k = 5, bs = "fs") +
## s(sample_id, bs = "re", k = 5) + s(subject, bs = "re", k = 5) +
## s(class_nb, k = 5) + s(total_reads_orig, k = 5)
##
## Parametric coefficients:
## Estimate Std. Error z value Pr(>|z|)
## (Intercept) -7.41616 0.06345 -116.9 <2e-16 ***
## ---
## Signif. codes: 0 '***' 0.001 '**' 0.01 '*' 0.05 '.' 0.1 ' ' 1
##
## Approximate significance of smooth terms:
## edf Ref.df Chi.sq p-value
## s(species,class_nb) 98.6016 336.000 3365.620 <2e-16 ***
## s(sample_id) 0.1877 464.000 0.149 0.9978
## s(subject) 145.5007 248.000 466.554 <2e-16 ***
## s(class_nb) 1.0027 1.005 5.712 0.0171 *
## s(total_reads_orig) 2.9709 3.353 108.694 <2e-16 ***
## ---
## Signif. codes: 0 '***' 0.001 '**' 0.01 '*' 0.05 '.' 0.1 ' ' 1
##
## R-sq.(adj) = 0.244 Deviance explained = 32.8%
## -REML = -56567 Scale est. = 1 n = 8324

### 6.3. Polymorphism rate (NS) as a function of order richness

##
## Family: Beta regression(846.954)
## Link function: logit
##
## Formula:
## polymorphism_nonsyn ~ s(species, order_nb, bs = "fs") + s(sample_id,
## bs = "re") + s(subject, bs = "re") + s(order_nb) + s(total_reads_orig)
##
## Parametric coefficients:
## Estimate Std. Error z value Pr(>|z|)
## (Intercept) -7.42185 0.06436 -115.3 <2e-16 ***
## ---
## Signif. codes: 0 '***' 0.001 '**' 0.01 '*' 0.05 '.' 0.1 ' ' 1
##
## Approximate significance of smooth terms:
## edf Ref.df Chi.sq p-value
## s(species,order_nb) 68.9809 658.000 3269.251 < 2e-16 ***
## s(sample_id) 0.1326 464.000 0.108 0.994300
## s(subject) 145.7224 248.000 461.058 < 2e-16 ***
## s(order_nb) 1.0352 1.059 11.954 0.000611 ***
## s(total_reads_orig) 3.3771 4.074 108.737 < 2e-16 ***
## ---
## Signif. codes: 0 '***' 0.001 '**' 0.01 '*' 0.05 '.' 0.1 ' ' 1
##
## R-sq.(adj) = 0.244 Deviance explained = 32.4%
## -REML = -56588 Scale est. = 1 n = 8324

### 6.4. Polymorphism rate (NS) as a function of family richness

##
## Family: Beta regression(844.979)
## Link function: logit
##
## Formula:
## polymorphism_nonsyn ~ s(species, family_nb, bs = "fs") + s(sample_id,
## bs = "re") + s(subject, bs = "re") + s(family_nb) + s(total_reads_orig)
##
## Parametric coefficients:
## Estimate Std. Error z value Pr(>|z|)
## (Intercept) -7.41469 0.05678 -130.6 <2e-16 ***
## ---
## Signif. codes: 0 '***' 0.001 '**' 0.01 '*' 0.05 '.' 0.1 ' ' 1
##
## Approximate significance of smooth terms:
## edf Ref.df Chi.sq p-value
## s(species,family_nb) 107.4280 664.000 3185.198 <2e-16 ***
## s(sample_id) 0.1441 464.000 0.118 0.991
## s(subject) 140.3559 248.000 419.052 <2e-16 ***
## s(family_nb) 1.5716 1.875 2.211 0.241
## s(total_reads_orig) 3.4335 4.134 108.800 <2e-16 ***
## ---
## Signif. codes: 0 '***' 0.001 '**' 0.01 '*' 0.05 '.' 0.1 ' ' 1
##
## R-sq.(adj) = 0.243 Deviance explained = 32.6%
## -REML = -56551 Scale est. = 1 n = 8324

### 6.5. Polymorphism rate (NS) as a function of genus richness

##
## Family: Beta regression(847.977)
## Link function: logit
##
## Formula:
## polymorphism_nonsyn ~ s(species, genus_nb, bs = "fs") + s(sample_id,
## bs = "re") + s(subject, bs = "re") + s(genus_nb) + s(total_reads_orig)
##
## Parametric coefficients:
## Estimate Std. Error z value Pr(>|z|)
## (Intercept) -7.40821 0.06158 -120.3 <2e-16 ***
## ---
## Signif. codes: 0 '***' 0.001 '**' 0.01 '*' 0.05 '.' 0.1 ' ' 1
##
## Approximate significance of smooth terms:
## edf Ref.df Chi.sq p-value
## s(species,genus_nb) 106.7698 661.000 3261.527 <2e-16 ***
## s(sample_id) 0.2152 464.000 0.177 0.991
## s(subject) 143.3437 248.000 444.411 <2e-16 ***
## s(genus_nb) 1.0310 1.052 2.434 0.122
## s(total_reads_orig) 3.4900 4.209 107.829 <2e-16 ***
## ---
## Signif. codes: 0 '***' 0.001 '**' 0.01 '*' 0.05 '.' 0.1 ' ' 1
##
## R-sq.(adj) = 0.243 Deviance explained = 32.9%
## -REML = -56559 Scale est. = 1 n = 8324

## 7. GLMMs with strain count in a focal species as a function of community diversity

### 7.1. Strain count in a focal species as a function of Shannon diversity

## Family: truncated_nbinom2 ( log )
## Formula:
## strain_nb ~ shannon_diversity + total_reads_orig + (1 | species_id)
## Data: datsc
##
## AIC BIC logLik deviance df.resid
## 25218.2 25256.4 -12604.1 25208.2 15350
##
## Random effects:
##
## Conditional model:
## Groups Name Variance Std.Dev.
## species_id (Intercept) 0.2825 0.5315
## Number of obs: 15355, groups: species_id, 184
##
## Overdispersion parameter for truncated_nbinom2 family (): 5.71e+05
##
## Conditional model:
## Estimate Std. Error z value Pr(>|z|)
## (Intercept) -0.30024 0.05086 -5.903 3.57e-09 ***
## shannon_diversity 0.05927 0.01178 5.033 4.83e-07 ***
## total_reads_orig 0.03362 0.01087 3.092 0.00199 **
## ---
## Signif. codes: 0 '***' 0.001 '**' 0.01 '*' 0.05 '.' 0.1 ' ' 1

### 7.2. Strain count in a focal species as a function of species richness (all data)

## Family: truncated_nbinom1 ( log )
## Formula:
## strain_nb ~ species_richness + total_reads_orig + (species_richness |
## species_id) + (1 | sample_id) + (1 | subject_id)
## Data: datsc
##
## AIC BIC logLik deviance df.resid
## 24936.4 25005.1 -12459.2 24918.4 15346
##
## Random effects:
##
## Conditional model:
## Groups Name Variance Std.Dev. Corr
## species_id (Intercept) 0.3005843 0.54826
## species_richness 0.0001944 0.01394 0.76
## sample_id (Intercept) 0.0218740 0.14790
## subject_id (Intercept) 0.0629320 0.25086
## Number of obs: 15355, groups: species_id, 184; sample_id, 467; subject_id, 249
##
## Overdispersion parameter for truncated_nbinom1 family (): 1.76e-05
##
## Conditional model:
## Estimate Std. Error z value Pr(>|z|)
## (Intercept) -0.32293 0.05560 -5.808 6.31e-09 ***
## species_richness -0.09799 0.02114 -4.636 3.56e-06 ***
## total_reads_orig 0.10792 0.02163 4.990 6.02e-07 ***
## ---
## Signif. codes: 0 '***' 0.001 '**' 0.01 '*' 0.05 '.' 0.1 ' ' 1

### 7.3. Strain count in a focal species as a function of richness (rarefied data)

## Family: truncated_nbinom2 ( log )
## Formula:
## strain_nb ~ species_richness + total_reads_orig + (1 | species_id)
## Data: datsc
##
## AIC BIC logLik deviance df.resid
## 24680.5 24718.5 -12335.2 24670.5 14958
##
## Random effects:
##
## Conditional model:
## Groups Name Variance Std.Dev.
## species_id (Intercept) 0.2917 0.5401
## Number of obs: 14963, groups: species_id, 180
##
## Overdispersion parameter for truncated_nbinom2 family (): 2.89e+05
##
## Conditional model:
## Estimate Std. Error z value Pr(>|z|)
## (Intercept) -0.28554 0.05171 -5.522 3.35e-08 ***
## species_richness -0.05129 0.01182 -4.341 1.42e-05 ***
## total_reads_orig 0.01634 0.01118 1.462 0.144
## ---
## Signif. codes: 0 '***' 0.001 '**' 0.01 '*' 0.05 '.' 0.1 ' ' 1

## 8. Strain count in a focal species as a function of shannon diversity at higher taxonomic levels

### 8.1. Strain count in a focal species as a function of phylum shannon diversity

## Family: truncated_nbinom2 ( log )
## Formula:
## strain_nb ~ phyla_shannon + total_reads_orig + (phyla_shannon |
## species_id) + (1 | sample_id) + (1 | subject_id)
## Data: datsc2
##
## AIC BIC logLik deviance df.resid
## 24949.9 25018.6 -12465.9 24931.9 15346
##
## Random effects:
##
## Conditional model:
## Groups Name Variance Std.Dev. Corr
## species_id (Intercept) 0.306800 0.55390
## phyla_shannon 0.001671 0.04087 -0.99
## sample_id (Intercept) 0.025953 0.16110
## subject_id (Intercept) 0.065625 0.25617
## Number of obs: 15355, groups: species_id, 184; sample_id, 467; subject_id, 249
##
## Overdispersion parameter for truncated_nbinom2 family (): 3.35e+04
##
## Conditional model:
## Estimate Std. Error z value Pr(>|z|)
## (Intercept) -0.32635 0.05658 -5.768 8.01e-09 ***
## phyla_shannon 0.03018 0.01951 1.547 0.12190
## total_reads_orig 0.06520 0.02062 3.162 0.00157 **
## ---
## Signif. codes: 0 '***' 0.001 '**' 0.01 '*' 0.05 '.' 0.1 ' ' 1

### 8.2. Strain count in a focal species as a function of class shannon diversity

## Family: truncated_nbinom2 ( log )
## Formula:
## strain_nb ~ class_shannon + total_reads_orig + (class_shannon |
## species_id) + (1 | sample_id) + (1 | subject_id)
## Data: datsc2
##
## AIC BIC logLik deviance df.resid
## 24949.7 25018.4 -12465.8 24931.7 15346
##
## Random effects:
##
## Conditional model:
## Groups Name Variance Std.Dev. Corr
## species_id (Intercept) 0.308816 0.5557
## class_shannon 0.001823 0.0427 -1.00
## sample_id (Intercept) 0.026762 0.1636
## subject_id (Intercept) 0.065165 0.2553
## Number of obs: 15355, groups: species_id, 184; sample_id, 467; subject_id, 249
##
## Overdispersion parameter for truncated_nbinom2 family (): 3.56e+04
##
## Conditional model:
## Estimate Std. Error z value Pr(>|z|)
## (Intercept) -0.32521 0.05670 -5.736 9.7e-09 ***
## class_shannon 0.03187 0.01956 1.629 0.10333
## total_reads_orig 0.06615 0.02067 3.201 0.00137 **
## ---
## Signif. codes: 0 '***' 0.001 '**' 0.01 '*' 0.05 '.' 0.1 ' ' 1

### 8.3. Strain count in a focal species as a function of order shannon diversity

## Family: truncated_nbinom2 ( log )
## Formula:
## strain_nb ~ order_shannon + total_reads_orig + (order_shannon |
## species_id)
## Data: datsc2
##
## AIC BIC logLik deviance df.resid
## 25235.2 25288.7 -12610.6 25221.2 15348
##
## Random effects:
##
## Conditional model:
## Groups Name Variance Std.Dev. Corr
## species_id (Intercept) 0.292881 0.54118
## order_shannon 0.002037 0.04513 -1.00
## Number of obs: 15355, groups: species_id, 184
##
## Overdispersion parameter for truncated_nbinom2 family (): 3.54e+09
##
## Conditional model:
## Estimate Std. Error z value Pr(>|z|)
## (Intercept) -0.29546 0.05197 -5.685 1.31e-08 ***
## order_shannon 0.03425 0.01359 2.521 0.0117 *
## total_reads_orig 0.02811 0.01092 2.575 0.0100 *
## ---
## Signif. codes: 0 '***' 0.001 '**' 0.01 '*' 0.05 '.' 0.1 ' ' 1

### 8.4. Strain count in a focal species as a function of family shannon diversity

## Family: truncated_nbinom2 ( log )
## Formula:
## strain_nb ~ family_shannon + total_reads_orig + (family_shannon |
## species_id) + (1 | sample_id) + (1 | subject_id)
## Data: datsc2
##
## AIC BIC logLik deviance df.resid
## 24952.5 25021.3 -12467.3 24934.5 15346
##
## Random effects:
##
## Conditional model:
## Groups Name Variance Std.Dev. Corr
## species_id (Intercept) 0.306320 0.5535
## family_shannon 0.001289 0.0359 -0.99
## sample_id (Intercept) 0.026419 0.1625
## subject_id (Intercept) 0.064846 0.2546
## Number of obs: 15355, groups: species_id, 184; sample_id, 467; subject_id, 249
##
## Overdispersion parameter for truncated_nbinom2 family (): 4e+04
##
## Conditional model:
## Estimate Std. Error z value Pr(>|z|)
## (Intercept) -0.32556 0.05635 -5.777 7.59e-09 ***
## family_shannon 0.02981 0.01966 1.516 0.12952
## total_reads_orig 0.06610 0.02062 3.206 0.00135 **
## ---
## Signif. codes: 0 '***' 0.001 '**' 0.01 '*' 0.05 '.' 0.1 ' ' 1

### 8.5. Strain count in a focal species as a function of genus shannon diversity

## Family: truncated_nbinom2 ( log )
## Formula:
## strain_nb ~ genus_shannon + total_reads_orig + (genus_shannon |
## species_id) + (1 | sample_id) + (1 | subject_id)
## Data: datsc2
##
## AIC BIC logLik deviance df.resid
## 24940.2 25008.9 -12461.1 24922.2 15346
##
## Random effects:
##
## Conditional model:
## Groups Name Variance Std.Dev. Corr
## species_id (Intercept) 0.311063 0.55773
## genus_shannon 0.003269 0.05717 -0.99
## sample_id (Intercept) 0.026939 0.16413
## subject_id (Intercept) 0.062524 0.25005
## Number of obs: 15355, groups: species_id, 184; sample_id, 467; subject_id, 249
##
## Overdispersion parameter for truncated_nbinom2 family (): 1.11e+08
##
## Conditional model:
## Estimate Std. Error z value Pr(>|z|)
## (Intercept) -0.32776 0.05672 -5.778 7.55e-09 ***
## genus_shannon 0.05746 0.02025 2.837 0.00455 **
## total_reads_orig 0.06556 0.02059 3.184 0.00145 **
## ---
## Signif. codes: 0 '***' 0.001 '**' 0.01 '*' 0.05 '.' 0.1 ' ' 1

## 9. GLMMs with strain count in a focal species as a function of richness at higher taxonomic levels

### 9.1. Strain count in a focal species as a function of phylum richness

## Family: truncated_nbinom2 ( log )
## Formula: strain_nb ~ phyla_nb + total_reads_orig + (1 | species_id)
## Data: datsc2
##
## AIC BIC logLik deviance df.resid
## 25226.1 25264.3 -12608.1 25216.1 15350
##
## Random effects:
##
## Conditional model:
## Groups Name Variance Std.Dev.
## species_id (Intercept) 0.2863 0.5351
## Number of obs: 15355, groups: species_id, 184
##
## Overdispersion parameter for truncated_nbinom2 family (): 1.95e+05
##
## Conditional model:
## Estimate Std. Error z value Pr(>|z|)
## (Intercept) -0.29144 0.05109 -5.705 1.17e-08 ***
## phyla_nb -0.04854 0.01145 -4.240 2.24e-05 ***
## total_reads_orig 0.03596 0.01102 3.262 0.0011 **
## ---
## Signif. codes: 0 '***' 0.001 '**' 0.01 '*' 0.05 '.' 0.1 ' ' 1

### 9.2. Strain count in a focal species as a function of class richness

## Family: truncated_nbinom1 ( log )
## Formula: strain_nb ~ class_nb + total_reads_orig + (1 | species_id) +
## (1 | sample_id) + (1 | subject_id)
## Data: datsc2
##
## AIC BIC logLik deviance df.resid
## 24941.6 24995.1 -12463.8 24927.6 15348
##
## Random effects:
##
## Conditional model:
## Groups Name Variance Std.Dev.
## species_id (Intercept) 0.30082 0.5485
## sample_id (Intercept) 0.02217 0.1489
## subject_id (Intercept) 0.06698 0.2588
## Number of obs: 15355, groups: species_id, 184; sample_id, 467; subject_id, 249
##
## Overdispersion parameter for truncated_nbinom1 family (): 1.26e-05
##
## Conditional model:
## Estimate Std. Error z value Pr(>|z|)
## (Intercept) -0.32627 0.05587 -5.840 5.23e-09 ***
## class_nb -0.06721 0.01819 -3.694 0.000221 ***
## total_reads_orig 0.08639 0.02075 4.163 3.14e-05 ***
## ---
## Signif. codes: 0 '***' 0.001 '**' 0.01 '*' 0.05 '.' 0.1 ' ' 1

### 9.3. Strain count in a focal species as a function of order richness

## Family: truncated_nbinom2 ( log )
## Formula:
## strain_nb ~ order_nb + total_reads_orig + (order_nb | species_id) +
## (1 | sample_id) + (1 | subject_id)
## Data: datsc2
##
## AIC BIC logLik deviance df.resid
## 24922.1 24990.8 -12452.0 24904.1 15346
##
## Random effects:
##
## Conditional model:
## Groups Name Variance Std.Dev. Corr
## species_id (Intercept) 0.298733 0.5466
## order_nb 0.001325 0.0364 0.99
## sample_id (Intercept) 0.021727 0.1474
## subject_id (Intercept) 0.059083 0.2431
## Number of obs: 15355, groups: species_id, 184; sample_id, 467; subject_id, 249
##
## Overdispersion parameter for truncated_nbinom2 family (): 2.16e+04
##
## Conditional model:
## Estimate Std. Error z value Pr(>|z|)
## (Intercept) -0.32528 0.05516 -5.897 3.71e-09 ***
## order_nb -0.11970 0.02012 -5.951 2.67e-09 ***
## total_reads_orig 0.11110 0.02127 5.224 1.75e-07 ***
## ---
## Signif. codes: 0 '***' 0.001 '**' 0.01 '*' 0.05 '.' 0.1 ' ' 1

### 9.4. Strain count in a focal species as a function of family richness

## Family: truncated_nbinom1 ( log )
## Formula:
## strain_nb ~ family_nb + total_reads_orig + (family_nb | species_id) +
## (1 | sample_id) + (1 | subject_id)
## Data: datsc2
##
## AIC BIC logLik deviance df.resid
## 24915.0 24983.7 -12448.5 24897.0 15346
##
## Random effects:
##
## Conditional model:
## Groups Name Variance Std.Dev. Corr
## species_id (Intercept) 0.2987158 0.54655
## family_nb 0.0005029 0.02242 0.89
## sample_id (Intercept) 0.0189422 0.13763
## subject_id (Intercept) 0.0590925 0.24309
## Number of obs: 15355, groups: species_id, 184; sample_id, 467; subject_id, 249
##
## Overdispersion parameter for truncated_nbinom1 family (): 1.71e-05
##
## Conditional model:
## Estimate Std. Error z value Pr(>|z|)
## (Intercept) -0.31927 0.05516 -5.788 7.11e-09 ***
## family_nb -0.12908 0.01937 -6.664 2.66e-11 ***
## total_reads_orig 0.11002 0.02038 5.398 6.73e-08 ***
## ---
## Signif. codes: 0 '***' 0.001 '**' 0.01 '*' 0.05 '.' 0.1 ' ' 1

### 9.5. Strain count in a focal species as a function of genus richness

## Family: truncated_nbinom1 ( log )
## Formula:
## strain_nb ~ genus_nb + total_reads_orig + (genus_nb | species_id) +
## (1 | sample_id) + (1 | subject_id)
## Data: datsc2
##
## AIC BIC logLik deviance df.resid
## 24943.1 25011.8 -12462.5 24925.1 15346
##
## Random effects:
##
## Conditional model:
## Groups Name Variance Std.Dev. Corr
## species_id (Intercept) 0.3011560 0.54878
## genus_nb 0.0001142 0.01069 0.86
## sample_id (Intercept) 0.0211474 0.14542
## subject_id (Intercept) 0.0671252 0.25909
## Number of obs: 15355, groups: species_id, 184; sample_id, 467; subject_id, 249
##
## Overdispersion parameter for truncated_nbinom1 family (): 1.82e-05
##
## Conditional model:
## Estimate Std. Error z value Pr(>|z|)
## (Intercept) -0.32475 0.05580 -5.820 5.90e-09 ***
## genus_nb -0.08379 0.02090 -4.009 6.09e-05 ***
## total_reads_orig 0.10105 0.02174 4.648 3.36e-06 ***
## ---
## Signif. codes: 0 '***' 0.001 '**' 0.01 '*' 0.05 '.' 0.1 ' ' 1

## 10. DBD over time in the gut (HMP time series)

### 10.1. Polymorphism change in a focal species as a function of community diversity at the earlier time point

#### 10.1.1. Polymorphism change in a focal species as a function of Shannon diversity at the earlier time point

##
## Family: gaussian
## Link function: identity
##
## Formula:
## log.delta_pi ~ s(total_reads_orig1) + s(alpha_div_tp1) + s(species,
## alpha_div_tp1, bs = "fs") + s(sample_tp1, bs = "re") + s(subject,
## bs = "re")
##
## Parametric coefficients:
## Estimate Std. Error t value Pr(>|t|)
## (Intercept) -4.943e-06 7.213e-05 -0.069 0.945
##
## Approximate significance of smooth terms:
## edf Ref.df F p-value
## s(total_reads_orig1) 1.000e+00 1.000 1.236 0.266297
## s(alpha_div_tp1) 2.286e+00 2.757 1.365 0.376384
## s(species,alpha_div_tp1) 2.099e-04 591.000 0.000 0.990102
## s(sample_tp1) 4.552e+01 215.000 0.297 0.000338 ***
## s(subject) 8.611e-04 159.000 0.000 0.721101
## ---
## Signif. codes: 0 '***' 0.001 '**' 0.01 '*' 0.05 '.' 0.1 ' ' 1
##
## R-sq.(adj) = 0.0227 Deviance explained = 3.83%
## GCV = 1.2171e-05 Scale est. = 1.1973e-05 n = 3063

#### 10.1.2. Polymorphism change in a focal species as a function of species richness at the earlier time point

##
## Family: gaussian
## Link function: identity
##
## Formula:
## log.delta_pi ~ s(total_reads_orig1) + s(richness_tp1) + s(species,
## richness_tp1, bs = "fs") + s(sample_tp1, bs = "re") + s(subject,
## bs = "re")
##
## Parametric coefficients:
## Estimate Std. Error t value Pr(>|t|)
## (Intercept) -1.072e-05 7.415e-05 -0.145 0.885
##
## Approximate significance of smooth terms:
## edf Ref.df F p-value
## s(total_reads_orig1) 1.000e+00 1.000 1.666 0.196889
## s(richness_tp1) 1.361e+00 1.585 0.256 0.606808
## s(species,richness_tp1) 1.051e-04 583.000 0.000 0.999966
## s(sample_tp1) 5.395e+01 215.000 0.357 0.000178 ***
## s(subject) 1.119e-04 159.000 0.000 0.821816
## ---
## Signif. codes: 0 '***' 0.001 '**' 0.01 '*' 0.05 '.' 0.1 ' ' 1
##
## R-sq.(adj) = 0.025 Deviance explained = 4.3%
## GCV = 1.2172e-05 Scale est. = 1.1945e-05 n = 3063

#### 10.1.3. Polymorphism change in a focal species as a function of the rarefied species richness at the earlier time point

##
## Family: gaussian
## Link function: identity
##
## Formula:
## log.delta_pi ~ s(richness_rare_tp1) + s(total_reads_orig) + s(species,
## richness_rare_tp1, bs = "fs") + s(sample_tp1, bs = "re") +
## s(subject, bs = "re")
##
## Parametric coefficients:
## Estimate Std. Error t value Pr(>|t|)
## (Intercept) -9.546e-06 7.241e-05 -0.132 0.895
##
## Approximate significance of smooth terms:
## edf Ref.df F p-value
## s(richness_rare_tp1) 1.000025 1.000 0.319 0.572048
## s(total_reads_orig) 1.005210 1.009 0.971 0.324110
## s(species,richness_rare_tp1) 4.108641 558.000 0.009 0.215833
## s(sample_tp1) 46.996360 215.000 0.312 0.000169 ***
## s(subject) 0.001492 159.000 0.000 0.711795
## ---
## Signif. codes: 0 '***' 0.001 '**' 0.01 '*' 0.05 '.' 0.1 ' ' 1
##
## R-sq.(adj) = 0.0241 Deviance explained = 4.1%
## GCV = 1.2171e-05 Scale est. = 1.1956e-05 n = 3063

### 10.2. Gene gain in a focal species as a function of community diversity at the earlier time point

#### 10.2.1. Gene gain in a focal species as a function of Shannon diversity at the earlier time point

## Family: nbinom1 ( log )
## Formula:
## num_gene_gains ~ alpha_div_tp1 + total_reads_orig1 + (alpha_div_tp1 |
## species) + (1 | subject) + (1 | sample_tp1)
## Data: datsc
##
## AIC BIC logLik deviance df.resid
## 1516.0 1562.5 -749.0 1498.0 1287
##
## Random effects:
##
## Conditional model:
## Groups Name Variance Std.Dev. Corr
## species (Intercept) 0.28885 0.5374
## alpha_div_tp1 0.08704 0.2950 -0.37
## subject (Intercept) 2.12132 1.4565
## sample_tp1 (Intercept) 1.00077 1.0004
## Number of obs: 1296, groups: species, 54; subject, 154; sample_tp1, 210
##
## Overdispersion parameter for nbinom1 family (): 121
##
## Conditional model:
## Estimate Std. Error z value Pr(>|z|)
## (Intercept) -0.36000 0.41245 -0.873 0.383
## alpha_div_tp1 0.07365 0.21687 0.340 0.734
## total_reads_orig1 -0.08165 0.23305 -0.350 0.726

#### 10.2.2. Gene gain in a focal species as a function of species richness at the earlier time point

## Family: nbinom1 ( log )
## Formula:
## num_gene_gains ~ richness_tp1 + total_reads_orig1 + (1 | species) +
## (1 | subject) + (1 | sample_tp1)
## Data: datsc
##
## AIC BIC logLik deviance df.resid
## 1513.1 1549.2 -749.5 1499.1 1289
##
## Random effects:
##
## Conditional model:
## Groups Name Variance Std.Dev.
## species (Intercept) 0.2914 0.5398
## subject (Intercept) 2.1331 1.4605
## sample_tp1 (Intercept) 0.8869 0.9418
## Number of obs: 1296, groups: species, 54; subject, 154; sample_tp1, 210
##
## Overdispersion parameter for nbinom1 family (): 123
##
## Conditional model:
## Estimate Std. Error z value Pr(>|z|)
## (Intercept) -0.28956 0.39887 -0.726 0.468
## richness_tp1 0.09735 0.19348 0.503 0.615
## total_reads_orig1 -0.14904 0.24677 -0.604 0.546

#### 10.2.3. Gene gain in a focal species as a function of rarefied richness at the earlier time point

## Family: nbinom1 ( log )
## Formula:
## num_gene_gains ~ richness_rare_tp1 + (1 | species) + (1 | subject) +
## (1 | sample_tp1)
## Data: datsc
##
## AIC BIC logLik deviance df.resid
## 1511.1 1542.1 -749.5 1499.1 1290
##
## Random effects:
##
## Conditional model:
## Groups Name Variance Std.Dev.
## species (Intercept) 0.2925 0.5408
## subject (Intercept) 2.1466 1.4651
## sample_tp1 (Intercept) 0.9015 0.9495
## Number of obs: 1296, groups: species, 54; subject, 154; sample_tp1, 210
##
## Overdispersion parameter for nbinom1 family (): 123
##
## Conditional model:
## Estimate Std. Error z value Pr(>|z|)
## (Intercept) -0.2833 0.3971 -0.713 0.476
## richness_rare_tp1 0.1127 0.1691 0.666 0.505

### 10.3. Gene loss in a focal species as a function of community diversity at the earlier time point

#### 10.3.1. Gene loss in a focal species as a function of Shannon diversity at the earlier time point

## Family: nbinom2 ( log )
## Formula:
## num_gene_losses ~ alpha_div_tp1 + total_reads_orig1 + (alpha_div_tp1 |
## species) + (1 | subject) + (1 | sample_tp1)
## Data: datsc
##
## AIC BIC logLik deviance df.resid
## 1696.2 1742.7 -839.1 1678.2 1287
##
## Random effects:
##
## Conditional model:
## Groups Name Variance Std.Dev. Corr
## species (Intercept) 1.7511 1.3233
## alpha_div_tp1 0.2719 0.5214 -0.57
## subject (Intercept) 2.3094 1.5197
## sample_tp1 (Intercept) 10.3411 3.2158
## Number of obs: 1296, groups: species, 54; subject, 154; sample_tp1, 210
##
## Overdispersion parameter for nbinom2 family (): 0.111
##
## Conditional model:
## Estimate Std. Error z value Pr(>|z|)
## (Intercept) -3.5455 0.8326 -4.259 2.06e-05 ***
## alpha_div_tp1 0.8360 0.3950 2.117 0.0343 *
## total_reads_orig1 0.8956 0.5299 1.690 0.0910 .
## ---
## Signif. codes: 0 '***' 0.001 '**' 0.01 '*' 0.05 '.' 0.1 ' ' 1

#### 10.3.2. Gene loss in a focal species as a function of species richness at the earlier time point

## Family: nbinom2 ( log )
## Formula:
## num_gene_losses ~ richness_tp1 + total_reads_orig1 + (1 | species) +
## (1 | subject) + (1 | sample_tp1)
## Data: datsc
##
## AIC BIC logLik deviance df.resid
## 1695.1 1731.2 -840.5 1681.1 1289
##
## Random effects:
##
## Conditional model:
## Groups Name Variance Std.Dev.
## species (Intercept) 1.577 1.256
## subject (Intercept) 3.432 1.852
## sample_tp1 (Intercept) 8.125 2.850
## Number of obs: 1296, groups: species, 54; subject, 154; sample_tp1, 210
##
## Overdispersion parameter for nbinom2 family (): 0.102
##
## Conditional model:
## Estimate Std. Error z value Pr(>|z|)
## (Intercept) -3.3062 0.7822 -4.227 2.37e-05 ***
## richness_tp1 0.7851 0.3751 2.093 0.0364 *
## total_reads_orig1 0.3357 0.5010 0.670 0.5028
## ---
## Signif. codes: 0 '***' 0.001 '**' 0.01 '*' 0.05 '.' 0.1 ' ' 1

#### 10.3.3. Gene loss in a focal species as a function of rarefied richness at the earlier time point

## Family: nbinom1 ( log )
## Formula:
## num_gene_losses ~ richness_rare_tp1 + total_reads_orig1 + (richness_rare_tp1 |
## species) + (1 | subject) + (1 | sample_tp1)
## Data: datsc
##
## AIC BIC logLik deviance df.resid
## 1709.6 1756.1 -845.8 1691.6 1287
##
## Random effects:
##
## Conditional model:
## Groups Name Variance Std.Dev. Corr
## species (Intercept) 0.22747 0.4769
## richness_rare_tp1 0.09539 0.3088 -0.58
## subject (Intercept) 0.94132 0.9702
## sample_tp1 (Intercept) 1.01547 1.0077
## Number of obs: 1296, groups: species, 54; subject, 154; sample_tp1, 210
##
## Overdispersion parameter for nbinom1 family (): 175
##
## Conditional model:
## Estimate Std. Error z value Pr(>|z|)
## (Intercept) 0.4533 0.3213 1.411 0.1583
## richness_rare_tp1 0.3501 0.1708 2.050 0.0404 *
## total_reads_orig1 0.2109 0.1827 1.155 0.2483
## ---
## Signif. codes: 0 '***' 0.001 '**' 0.01 '*' 0.05 '.' 0.1 ' ' 1

## 11. DBD over time in the gut (Poyet time series)

### 11.1 polymorphism rate variation as a function of Shannon diversity at the earlier time point

##
## Family: gaussian
## Link function: identity
##
## Formula:
## log.delta_pi ~ ti(alpha_div_tp1, lag) + s(read_count) + s(species,
## bs = "re") + s(subject, bs = "re") + s(sample_tp1, bs = "re") +
## s(sample_tp2, bs = "re")
##
## Parametric coefficients:
## Estimate Std. Error t value Pr(>|t|)
## (Intercept) 0.0003851 0.0006152 0.626 0.531
##
## Approximate significance of smooth terms:
## edf Ref.df F p-value
## ti(alpha_div_tp1,lag) 5.402 6.915 2.325 0.0234 *
## s(read_count) 1.006 1.006 1.633 0.1998
## s(species) 13.552 14.000 3179.569 2.83e-07 ***
## s(subject) 1.897 3.000 93229.029 0.2388
## s(sample_tp1) 352.188 362.000 364.322 3.55e-16 ***
## s(sample_tp2) 350.584 359.000 2950.333 5.61e-15 ***
## ---
## Signif. codes: 0 '***' 0.001 '**' 0.01 '*' 0.05 '.' 0.1 ' ' 1
##
## R-sq.(adj) = 0.655 Deviance explained = 66.3%
## -ML = -1.6937e+05 Scale est. = 1.3729e-06 n = 32113

### 11.2 polymorphism rate variation as a function of rarefied richness at the earlier time point

##
## Family: gaussian
## Link function: identity
##
## Formula:
## log.delta_pi ~ ti(richness_rare_tp1, lag) + s(read_count) + s(species,
## bs = "re") + s(subject, bs = "re") + s(sample_tp1, bs = "re") +
## s(sample_tp2, bs = "re")
##
## Parametric coefficients:
## Estimate Std. Error t value Pr(>|t|)
## (Intercept) 0.0004998 0.0005718 0.874 0.382
##
## Approximate significance of smooth terms:
## edf Ref.df F p-value
## ti(richness_rare_tp1,lag) 0.01718 16 0.001 0.689
## s(read_count) 0.31775 9 4909.967 0.225
## s(species) 13.54785 14 2995.679 6.36e-10 ***
## s(subject) 0.02014 3 28.740 0.290
## s(sample_tp1) 353.84479 363 1114.032 1.31e-06 ***
## s(sample_tp2) 351.65462 359 6153.476 3.90e-07 ***
## ---
## Signif. codes: 0 '***' 0.001 '**' 0.01 '*' 0.05 '.' 0.1 ' ' 1
##
## R-sq.(adj) = 0.655 Deviance explained = 66.3%
## -ML = -1.6936e+05 Scale est. = 1.3735e-06 n = 32113

### 11.3 Gene gain in a focal species as a function of Shannon diversity at the earlier time point

## Family: nbinom2 ( log )
## Formula:
## num_gene_gains ~ alpha_div_tp1 * lag + read_count + (1 | species) +
## (1 | subject) + (1 | sample_tp1) + (1 | sample_tp2)
## Data: datsc
##
## AIC BIC logLik deviance df.resid
## 13058.3 13142.0 -6519.1 13038.3 32103
##
## Random effects:
##
## Conditional model:
## Groups Name Variance Std.Dev.
## species (Intercept) 4.8180 2.195
## subject (Intercept) 3.8308 1.957
## sample_tp1 (Intercept) 0.5373 0.733
## sample_tp2 (Intercept) 6.3800 2.526
## Number of obs: 32113, groups:
## species, 15; subject, 4; sample_tp1, 364; sample_tp2, 360
##
## Overdispersion parameter for nbinom2 family (): 2.54
##
## Conditional model:
## Estimate Std. Error z value Pr(>|z|)
## (Intercept) -5.86584 1.32874 -4.415 1.01e-05 ***
## alpha_div_tp1 -0.50462 0.07201 -7.008 2.42e-12 ***
## lag 0.64204 0.06529 9.834 < 2e-16 ***
## read_count -0.06774 0.05990 -1.131 0.258
## alpha_div_tp1:lag -0.21991 0.03632 -6.054 1.41e-09 ***
## ---
## Signif. codes: 0 '***' 0.001 '**' 0.01 '*' 0.05 '.' 0.1 ' ' 1

### 11.4 Gene gain in a focal species as a function of rarefied richness at the earlier time point

## Family: nbinom2 ( log )
## Formula: num_gene_gains ~ richness_rare_tp1 * lag + read_count + (1 |
## species) + (1 | subject) + (1 | sample_tp1) + (1 | sample_tp2)
## Data: datsc1
##
## AIC BIC logLik deviance df.resid
## 13120.8 13204.5 -6550.4 13100.8 32103
##
## Random effects:
##
## Conditional model:
## Groups Name Variance Std.Dev.
## species (Intercept) 5.1570 2.2709
## subject (Intercept) 3.6070 1.8992
## sample_tp1 (Intercept) 0.6188 0.7866
## sample_tp2 (Intercept) 6.2895 2.5079
## Number of obs: 32113, groups:
## species, 15; subject, 4; sample_tp1, 364; sample_tp2, 360
##
## Overdispersion parameter for nbinom2 family (): 2.41
##
## Conditional model:
## Estimate Std. Error z value Pr(>|z|)
## (Intercept) -6.13441 1.32483 -4.630 3.65e-06 ***
## richness_rare_tp1 -0.05682 0.06514 -0.872 0.383100
## lag 0.71254 0.06831 10.431 < 2e-16 ***
## read_count -0.17279 0.06142 -2.813 0.004908 **
## richness_rare_tp1:lag -0.13105 0.03661 -3.580 0.000343 ***
## ---
## Signif. codes: 0 '***' 0.001 '**' 0.01 '*' 0.05 '.' 0.1 ' ' 1

### 11.5 Gene loss in a focal species as a function of Shannon diversity at the earlier time point

## Family: nbinom2 ( log )
## Formula:
## num_gene_losses ~ alpha_div_tp1 * lag + read_count + (1 | species) +
## (1 | subject) + (1 | sample_tp1) + (1 | sample_tp2)
## Data: datsc
##
## AIC BIC logLik deviance df.resid
## 13168.6 13252.4 -6574.3 13148.6 32103
##
## Random effects:
##
## Conditional model:
## Groups Name Variance Std.Dev.
## species (Intercept) 1.7682 1.3297
## subject (Intercept) 3.0006 1.7322
## sample_tp1 (Intercept) 4.6178 2.1489
## sample_tp2 (Intercept) 0.3656 0.6047
## Number of obs: 32113, groups:
## species, 15; subject, 4; sample_tp1, 364; sample_tp2, 360
##
## Overdispersion parameter for nbinom2 family (): 3.8
##
## Conditional model:
## Estimate Std. Error z value Pr(>|z|)
## (Intercept) -6.32440 1.05926 -5.971 2.36e-09 ***
## alpha_div_tp1 0.81713 0.16397 4.984 6.24e-07 ***
## lag 0.22256 0.04946 4.500 6.79e-06 ***
## read_count -0.07615 0.15416 -0.494 0.62132
## alpha_div_tp1:lag 0.10992 0.04017 2.736 0.00621 **
## ---
## Signif. codes: 0 '***' 0.001 '**' 0.01 '*' 0.05 '.' 0.1 ' ' 1

### 11.6 Gene loss in a focal species as a function of rarefied richness at the earlier time point

## Family: nbinom2 ( log )
## Formula:
## num_gene_losses ~ richness_rare_tp1 * lag + read_count + (1 |
## species) + (1 | subject) + (1 | sample_tp1) + (1 | sample_tp2)
## Data: datsc1
##
## AIC BIC logLik deviance df.resid
## 13188.7 13272.4 -6584.3 13168.7 32103
##
## Random effects:
##
## Conditional model:
## Groups Name Variance Std.Dev.
## species (Intercept) 1.6549 1.2864
## subject (Intercept) 3.3111 1.8196
## sample_tp1 (Intercept) 4.9436 2.2234
## sample_tp2 (Intercept) 0.3674 0.6061
## Number of obs: 32113, groups:
## species, 15; subject, 4; sample_tp1, 364; sample_tp2, 360
##
## Overdispersion parameter for nbinom2 family (): 3.8
##
## Conditional model:
## Estimate Std. Error z value Pr(>|z|)
## (Intercept) -5.89552 1.07721 -5.473 4.43e-08 ***
## richness_rare_tp1 0.49358 0.16024 3.080 0.00207 **
## lag 0.21302 0.05107 4.171 3.03e-05 ***
## read_count 0.04467 0.15553 0.287 0.77395
## richness_rare_tp1:lag 0.04897 0.04520 1.083 0.27860
## ---
## Signif. codes: 0 '***' 0.001 '**' 0.01 '*' 0.05 '.' 0.1 ' ' 1
